# Monocular Depth Estimation Using Deep Learning: A Review

**DOI:** 10.3390/s22145353

**Published:** 2022-07-18

**Authors:** Armin Masoumian, Hatem A. Rashwan, Julián Cristiano, M. Salman Asif, Domenec Puig

**Affiliations:** 1 Department of Computer Engineering and Mathematics, University of Rovira i Virgili, 43007 Tarragona, Spain; hatem.abdellatif@urv.cat (H.A.R.); julianefren.cristiano@urv.cat (J.C.); domenec.puig@urv.cat (D.P.); 2 Department of Electrical and Computer Engineering, University of California, Riverside, CA 92521, USA; sasif@ece.ucr.edu

**Keywords:** monocular depth estimation, single image depth estimation, deep learning, multi-task learning, supervised, semi-supervised, and unsupervised learning

## Abstract

In current decades, significant advancements in robotics engineering and autonomous vehicles have improved the requirement for precise depth measurements. Depth estimation (DE) is a traditional task in computer vision that can be appropriately predicted by applying numerous procedures. This task is vital in disparate applications such as augmented reality and target tracking. Conventional monocular DE (MDE) procedures are based on depth cues for depth prediction. Various deep learning techniques have demonstrated their potential applications in managing and supporting the traditional ill-posed problem. The principal purpose of this paper is to represent a state-of-the-art review of the current developments in MDE based on deep learning techniques. For this goal, this paper tries to highlight the critical points of the state-of-the-art works on MDE from disparate aspects. These aspects include input data shapes and training manners such as supervised, semi-supervised, and unsupervised learning approaches in combination with applying different datasets and evaluation indicators. At last, limitations regarding the accuracy of the DL-based MDE models, computational time requirements, real-time inference, transferability, input images shape and domain adaptation, and generalization are discussed to open new directions for future research.

## 1. Introduction

**Figure 1:**
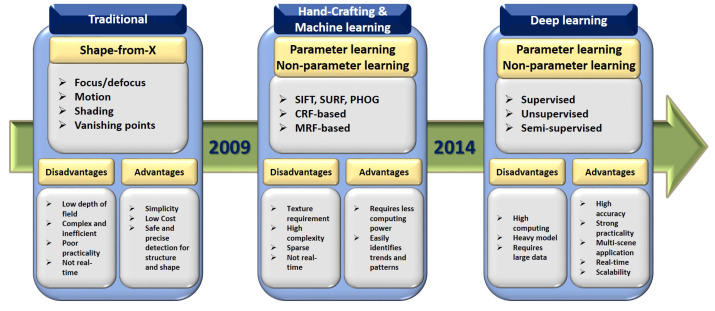
Evaluation trend of DE approaches divided into three sections: traditional methods, hand-crafting and machine learning methods, and deep learning methods.

Indisputable breakthroughs in the field of computational photography have helped the emergence of novel functionalities in the imaging process [1,2]. Many works have been carried out so far in the field of computer vision [3,4,5,6]. Depth estimation (DE) is a traditional computer vision task that predicts depth from one or more two-dimensional (2D) images. DE estimates each pixel’s depth in an image using offline-trained models. In machine perception, recognition of some functional factors such as the shape of a scene from an image and image independence from its appearance seems to be fundamental [7,8,9]. DE has great potential for use in disparate applications, including grasping in robotics, robot-assisted surgery, computer graphics, and computational photography [10,11,12,13,14,15]. Figure 1 schematically illustrates the evaluation trend of DE.

The DE task needs an RGB image and a depth image as output. The depth image often consists of data about the distance of the object in the image from the camera viewpoint [16]. The computer-based DE approach has been under evaluation by various investigators worldwide, and the DE problem has been an exciting field of research. Most successful computer-based methods are employed by determining depth by applying stereo vision. With the progress of recent deep learning (DL) models, DE based on DL models has been able to demonstrate its remarkable efficiency in many applications [17,18,19]. DE can be functionally classified into three divisions, including monocular depth estimation (MDE), binocular depth estimation (BDE), or multi-view depth estimation (MVDE).

MDE is an identified significant challenge in computer vision, in which no reliable cues exist to perceive depth from a single image. For instance, stereo correspondences are easily lost from MDE images [20]. Thus, the classical DE methods profoundly depend on multi-view geometry such as stereo images [21,22]. These approaches need alignment procedures, which are of great importance for stereo- or multi-camera depth measurement systems [23,24]. Consequently, using visual cues and disparate camera parameters, BDE and MVDE methods helps to obtain depth information (DI). The majority of BDE or MVDE techniques can accurately estimate DI; however, many practical/operational challenges, such as calculation time and memory requirements for different applications, should be considered [17,25]. The application of monocular images seems to be an excellent idea to capture DI to solve the memory requirement problem. The recent progression in using convolutional neural networks (CNN) and recurrent convolutional neural networks (RNN) yields a considerable improvement in the performance of MDE procedures [26,27,28].

Scientists worldwide have conducted various medical-based investigations to study the difference in depth perception with MDE or BDE systems. Despite the efforts to use BDE or MVDE systems to estimate depths up to hundreds of meters, the majority of results imply that the most efficient distance for a BDE system is restricted to almost 10 m [29,30,31]. Small baseline of stereo pairs is the main reason behind the small depth range. Beyond this amount, human vision follows a monocular situation [31]. According to this information, it is obvious that the MDE systems can make better depth predictions than a human. Some problems, including the requirement for a great amount of training data and domain adaptation issues, exist and must be solved appropriately [32].

In addition, research shows that industrial companies are looking at reducing costs and increasing the performance of their AI-based systems. Therefore, this article discusses the main advantages of MDE compared to stereo-based DE due to the low cost of grabbing sensors. In addition, it compares the MDE models from different aspects such as input data shapes and training manner. It discusses the advantages and disadvantages of each model to make it easier for the companies to better understand the differences between these models and select the suitable model for their system.

This paper aims to review the highlighted studies on the recent advancements in the functional application of deep-learning-based MDE. Thus, many DE works from different aspects, including data input types (mono-sequence [16,33,34], stereo sequence [7,26] and sequence-to-sequence [35,36]) and the training manner (i.e., supervised learning (SL) [9,37,38], unsupervised learning (UL) [16,39,40], and semi-supervised learning (SSL) [26,41,42] approaches) combined with the application of different datasets and evaluation indicators have been studied. Eventually, key points and future outlooks such as the accuracy, computational time, resolution quality, real-time inference, transferability, and input data shapes are discussed to open new horizons for future research.

This survey includes over 150 papers, most of them recent, on a wide variety of applications of DL in MDE. To identify relevant contributions, PubMed was queried for papers containing (“Depth Estimation” OR “Relative Distance Prediction”) in the title or abstract. ArXiv was searched for papers mentioning one of a set of terms related to computer vision. Additionally, conference proceedings for CVPR and ICCV were searched based on the titles of papers. We checked references in all selected papers and consulted colleagues. The papers without reported results are excluded. When overlapping work had been reported in multiple publications, only the publication(s) deemed most important were included.

Several surveys concerning MDE have been published in recent years, as summarized in Table 1. In this survey, we are concerned with six parameters that are used to assess any MDE method; “TM”: training manner, “ACC”: accuracy, “CT”: computational Time, “RQ”: resolution quality, “RTI”: real-time inference, “TRAN”: transferability, “IDS”: input data shapes. In Table 1, we also compare our paper to the recent surveys in terms of the six parameters to show that all of these surveys do not focus on all of these parameters.

This survey is organized in the following way: Section 2 describes the background of DE. The DE task’s main datasets and evaluation metrics are reviewed in Section 3 and Section 4, respectively. MDE based on DL models and a comparison of three main data input shapes and training manner approaches are described in Section 5 and Section 6. Section 7 presents the discussion, and Section 8 concludes this review.

## 2. Depth Estimation (DE)

Objects’ depth in a scene possesses the remarkable ability of estimation/calculation by applying passive and active approaches. In the active approaches (i.e., applications of LIDAR sensors and RGB-D cameras), the DI is achieved quickly [46,47]. RGB-D camera is a specific type of depth-sensing device that combines an RGB image and its corresponding depth image [48]. RGB-D cameras can be used in various devices such as smartphones and unmanned aerial systems due to their low cost and power consumption [49]. RGB-D cameras have limited depth range and they suffer from specular reflections and absorbing objects. Therefore, many depth completion approaches have been proposed to mitigate the gap between sparse and dense depth maps [44].

**Figure 2:**
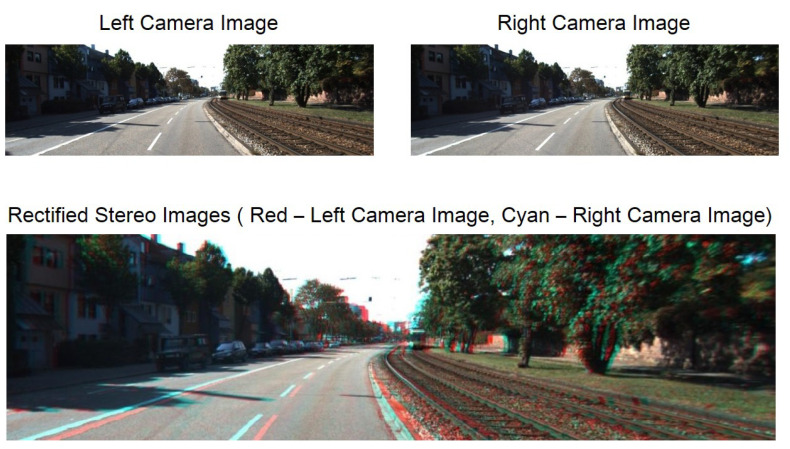
(**Top**) Non-rectified left and right images, and (**down**) red–cyan anaglyph from stereo pair of rectified stereo images.

In passive techniques, DI is often achieved using two principal methodologies: depth from stereo images and monocular images. The main purpose of both techniques is to assist in building the spatial structure of the environment, which presents a 3D view of the scene. After achieving DI, the situation of the viewer would be recognized relative to the surrounding objects. Stereo vision is a widely-applied depth calculation procedure in the computer vision area. Stereo vision is known as a computer-based passive approach in which stereo images are applied to extract DI [50,51,52]. To compute disparity, pixel matching must be implemented among the pixels of both images. It is worth noting that a good correspondence (pixels) matching needs the rectification of both images. Rectification is defined as the transformation process of images to match the epipolar lines of the original images horizontally [53,54]. Figure 2 demonstrates the images before and after the rectification process. The matching process of the pixel in an image with its similar pixel in another image along an epipolar line occurs using a matching cost function. By matching the pixels of both images, the calculation of depth applying the distance between two cameras and the pixel distance between matched pixels will be possible [55,56]. Reflective and highly transparent zones accompanied by smooth areas are the major challenges for stereo matching algorithms. Owing to perspective alteration, an image’s edge details can disappear in the second image. If the algorithm does not have sufficient capability to match the edge points on another image, it can create an erroneous depth value and noise in the predicted depth map at those points [57,58].

Sometimes, the application of algorithms for calculating depth may create different challenges. For instance, the matching cost function utilized in the algorithm can generate false-positive signals, which eventuates in the creation of depth maps with low accuracy. Thus, the use of post-processing approaches (i.e., median filter, bilateral filter, and interpolation) is of great importance in stereo vision applications to delete noise and refine depth maps [59,60,61,62].

**Figure 3:**
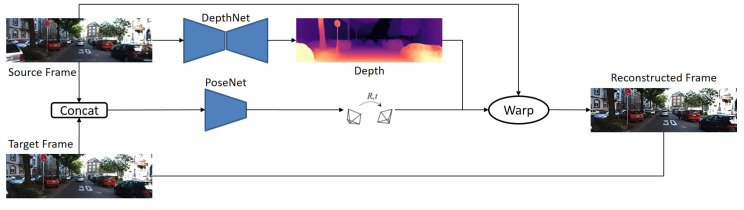
Main network structure for MDE [34]. This network contain two sub-networks: DepthNet for predicting the depth map and PoseNet for estimating the camera pose.

On the contrary, MDE does not require rectified images since MDE models work with a sequence of images extracted from a single camera. This simplicity and easy access are one of the main advantages of MDE compared to stereo models, which require additional complicated pieces of equipment. Because of that, in recent years, demand for MDE increased significantly. Most MDE methods focused on estimating distances between scene objects and the camera from one viewpoint. It is essential for regressing depth in 3D space in MDE methods since there is a lack of reliable stereoscopic visual relationship in which images adopt a 2D form to reflect the 3D space [15]. Therefore, MDE models try to recover the depth maps of images, which reflects the 3D structure of the scene. Most of the MDE models have the main architecture, which contains two main parts: depth and pose networks. The depth network predicts the depth maps. In turn, the pose network works as an ego-motion estimation (i.e., rotation and translation of the camera) between two successive images. The estimated depth (i.e., disparity) maps with the ego-motion parameters used to reconstruct an image should be compared to the target image. Figure 3 represents the schematic illustration of this method.

## 3. Datasets

There are various types of datasets for depth prediction based on different viewpoints. This section highlights the most popular public datasets of DL models for MDE.

### 3.1. KITTI

The KITTI dataset [63] is considered the most commonly applied dataset in computer vision, such as optical flow, visual odometry (VO), and semantic segmentation [63,64,65,66]. This dataset is also the most prevalent criterion in the unsupervised/semi-supervised MDE. In this dataset, 56 scenes are divided into two main compartments: 28 scenes for training and the rest for testing [9]. Due to the incredible capability of the KITTI dataset to create the pose ground truth for 11 odometry sequences, it is extensively applied to assess deep-learning-based VO algorithms [67,68]. This dataset contains 39,810 images for training, 4424 for validation, and 697 for testing. The resolution of the images is 1024×320 pixels. The MDE results of the UL, SL, and SSL procedures investigated on the KITTI dataset are presented in Table 2.

### 3.2. NYU Depth-V2

The NYU Depth [85] is a vital dataset, which includes 464 indoor scenes that concentrate on indoor environments. Compared to the KITTI dataset, which collects ground truth with LIDAR, this dataset accepts monocular video sequences of scenes and an RGB-D camera’s ground truth of depth. The NYU Depth is the main training dataset in the supervised MDE. The indoor scenes are divided into 249 and 215 sections for training and testing. Due to disparate variable frame rates, there is no one-to-one communication between depth maps and RGB images. Intending to arrange the depth and the RGB images, each depth map is related to the nearest RGB image. In addition, due to the discretion of the projection, all pixels do not possess an associated depth value. Therefore, those pixels that do not have depth value are masked within the experiments [28,85]. The resolution of the RGB images in sequences is 640×480 pixels. The MDE results of the investigation on the NYU-V2 dataset are presented in Table 3.

### 3.3. Cityscapes

This dataset prominently concentrates on semantic segmentation tasks. In this dataset, 5000 fine-annotation images and 20,000 coarse-annotations images exist [66,94]. Cityscapes dataset includes a series of stereo video sequences, which has only the potential of application for the training process of disparate unsupervised DE procedures [78]. The efficiency of depth networks can be significantly improved by pretraining the networks on the Cityscapes [7,16,95]. The training data of this dataset include 22,973 stereo image pairs with a resolution of 1024×2048.

### 3.4. Make3D

These data include both monocular RGB and depth images but do not possess stereo images that are different from the datasets mentioned above [96,97]. Due to the non-existence of monocular sequences in the Make3D dataset, SSL and UL procedures do not apply it as the training set, while SL techniques often adopt it for training. The fact of the matter is that the Make3D dataset is extensively used as a testing set of unsupervised algorithms to assess the production capability of networks on disparate datasets [7]. The RGB image resolution is 2272×1704, and the depth map resolution is 55×305 pixels. The MDE results of the investigation on the Make3D dataset are presented in Table 4.

### 3.5. DIODE

DIODE [103] is the Dense Indoor/Outdoor Depth dataset for monocular depth estimation comprising diverse indoor and outdoor scenes acquired with the same hardware setup. This dataset consists of 8574 indoor and 16,884 outdoor samples from 20 scans each for training and 325 indoor and 446 outdoor samples with each set from 10 different scans for validation with the resolution of 768×1024. The indoor and outdoor ranges for the dataset are 50 m and 300 m, respectively.

### 3.6. Middlebury 2014

Middlebury [104] is a dense indoor scene dataset which contains 33 images of 6-megapixel high resolution. Images are captured via two stereo DSLR cameras and two point-and-shoot cameras. Disparity ranges are between 200 and 800 pixels at a resolution of 6 megapixels. The image resolution of this dataset is 2872×1984.

### 3.7. Driving Stereo

The driving stereo [105] is one of the new large-scale stereo driving datasets that contains 182k images. The disparity images are captured via LIDAR, the same as the KITTI dataset. They mainly focus on two new metrics, a distance-aware metric and a semantic-aware metric, for evaluating stereo matching on MDE. The image resolution of this dataset is 1762×800. Table 5 represents the summary of datasets features for DE.

Although many valuable datasets and benchmarks exist for assessing monocular and stereo DE methods, there are still some limitations in the available datasets. For instance, all these datasets include images captured only during day or night, yet there are no datasets to have both together, and the same applies for indoor or outdoor images. In addition, no dataset concerns different challenges related to the change in weather conditions (e.g., fog, sunny, snow, etc.).

## 4. Evaluation Metrics

To assess the efficiency of the DE models, an accepted evaluation procedure was recommended by Eigen et al. [9], which possesses five evaluation metrics, including absolute relative difference (Abs-Rel), square relative error (Sq-Rel), root mean square error (RMSE), RMSE-log, and accuracy, with a threshold (δt). They are formulated using the following equations [9]:

(1) $$Abs-Rel=\frac{1}{\left|D\right|}\sum_{pred\;\in \;D}\;\left|gt-pred\right|/gt$$

(2) $$Sq-Rel=\frac{1}{\left|D\right|}\sum_{pred\;\in \;D}\;||gt-{pred||}^{2}/gt$$

(3) $$RMSE=\sqrt{\frac{1}{\left|D\right|}\sum_{pred\;\in \;D}\;||gt-{pred||}^{2}}$$

(4) $$RMSE-Log=\sqrt{\frac{1}{\left|D\right|}\sum_{pred\;\in \;D}\;||\log\left(gt\right)-{\log\left(pred\right)||}^{2}}$$

(5) $$\delta t=\frac{1}{\left|D\right|}\left|\left\{pred\;\in \;D\;|max\left(\frac{gt}{pred},\frac{pred}{gt}\right)<1.25^{t}\right\}\right|\times 100\%$$

In these equations, the pred and gt denote predicted depth and ground truth, respectively. D represents the set of all predicted depths value for a single image, |.| returns the number of the elements in each input set, and **δt** represents the threshold.

## 5. Input Data Shapes for MDE Applying Deep Learning

This section mainly introduces common types of data input for MDE. The input data shapes in MDE networks can be divided into three main categories: mono-sequence, stereo sequence, and sequence-to-sequence input data. Based on the architecture of the networks, the input data shapes will be different.

### 5.1. Mono-Sequence

**Figure 4:**
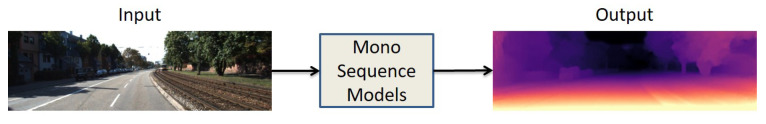
Data input/output structure of mono-sequence models. Single image input and single image output.

Monocular sequence input is mainly used for training the UL models. Figure 4 shows the basic structure of mono-sequence models, which have a single input image and a single output image. UL networks consist of a depth network for predicting depth maps and a pose network for camera pose estimation. The camera pose estimation works similarly to image transformation estimation, which helps to improve the results of MDE. These two sub-networks are connected in parallel, and the whole model is obliged to reconstruct the image. In mono-sequence, mostly the geometric constraints are built on adjacent frames. Lately, researchers have used VO [107] to predict the camera motion for learning the scene depth. Zhou et al. [16] were the pioneers of mono-sequence input type, and they proposed a network to predict camera motion and depth maps with photometric consistency loss and reconstruction loss.

**Figure 5:**
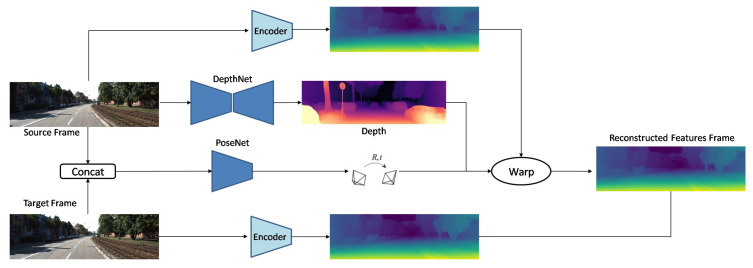
Developed network by Shu et al. [84].

Furthermore, Mahjourian et al. [33] introduced a network with 3D geometric constraints and enforced consistency of the estimated 3D point clouds and ego-motion across consecutive frames. Recently, Masoumian et al. [34] designed two jointly connected sub-networks for depth prediction and ego-motion. They used CNN-GCN encoder–decoder architecture for their networks with three losses: reconstruction loss, photometric loss, and smooth loss. In addition, Shu et al. [84] proposed a similar method with two jointly connected depth and pose predictions that were slightly different. They also added a feature extractor encoder to their model to improve the quality of their predicted depth maps. Their proposed architecture is shown in Figure 5.

### 5.2. Stereo Sequence

**Figure 6:**
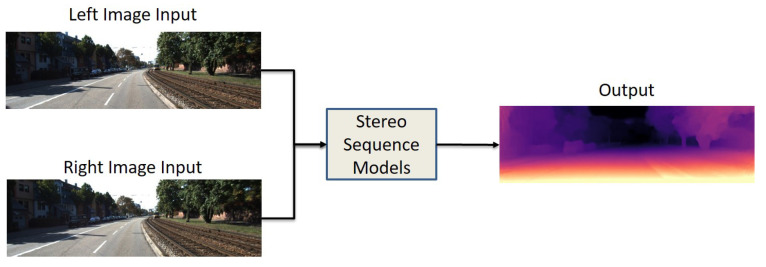
Data input/output structure of stereo sequence models. Stereo pairs of images as an input and single image output.

The projection and mapping relationship between the left and right pairwise images is mainly constrained by stereo matching. In order to build geometric constraints, a stereo images dataset is required. These types of inputs are commonly used in UL and SL networks. Figure 6 represents the basic structure of stereo sequence models which have left and right images as input and a single output. Similar to the monocular sequence input data shape, the stereo sequence works with image reconstruction with slight differences. An image will be reconstructed based on warping between the depth map and the right image. For instance, Kuznietsov et al. [26] proposed an SSL model for MDE with sparse data, and they built a stereo alignment as a geometric constraint.

**Figure 7:**
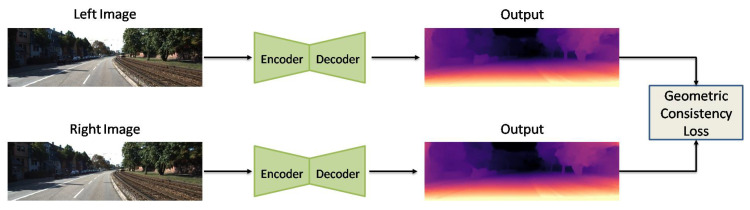
Developed network by Goldman et al. [108].

Furthermore, Godard et al. [7] designed a UL network with left–right consistency constraints. They used CNN-based encoder–decoder architecture for their model with the reconstruction loss, left–right disparity consistency, and disparity smoothness loss. Recently, Goldman et al. [108] proposed a Siamese network architecture with weight sharing, which consists of two twin networks, each learning to predict a disparity map from a single image. Their network is composed of an encoder–decoder pair with skip connections, which is shown in Figure 7.

### 5.3. Sequence-to-Sequence

**Figure 8:**
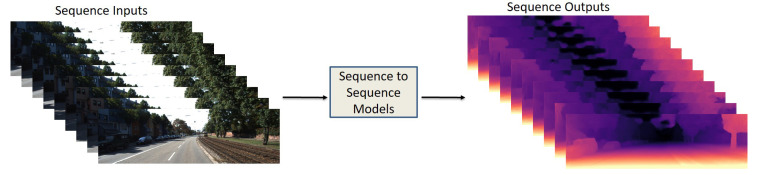
Data input/output structure of sequence-to-sequence models. Sequence of images as an input and sequence of images as an output.

**Figure 9:**
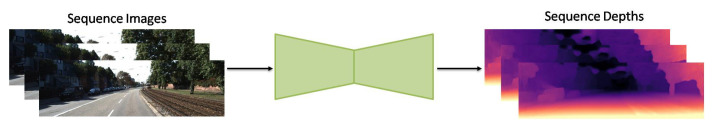
Developed network by Kumar et al. [35].

Sequence-to-sequence data input is necessary for recurrent neural network (RNN) models [109]. These models have memory capability, which helps the system learn a group of features in sequence images. Figure 8 represents the basic structure of sequence-to-sequence models, which have a sequence of images as input and a sequence of depth maps as an output. Most RNN methods use long short-term memory (LSTM) to learn the long-term dependencies with a three-gate structure [109]. However, RNN and CNN networks will be combined to extract spatial–temporal features. The sequence-to-sequence data primarily will be trained on SL models. Kumar et al. [35] proposed an MDE model with ConvLSTM layers for learning the smooth temporal variation. Their model consists of encoder–decoder architecture, which is shown in Figure 9. Furthermore, Mancini et al. [36] improved LSTM layers to obtain the best outcome of the predicted depth maps by feeding the input images sequentially to the system.

## 6. Mde Applying Deep Learning Training Manners

Although DE from multiple images possesses a lengthy background in the computer vision area, the DI extraction process from single images is considered a novel concept in DL. The advancements have initiated comprehensive investigations of the DI concept in DL techniques. The most critical challenge towards the application of DL is the absence of datasets that fit the problem [110,111,112]. This challenge may also be of great importance for the MDE network. Data applied in training may be collected by LIDAR sensors, RGB-D cameras, or stereo vision cameras. Despite the expensive data collection process, disparate learning strategies have been developed to decrease dependency on the dataset used for training. The learning process in MDE networks can be divided into three parts, including SL, UL, and SSL [7,9,26,37,40,113].

### 6.1. Supervised Learning Approach

**Figure 10:**
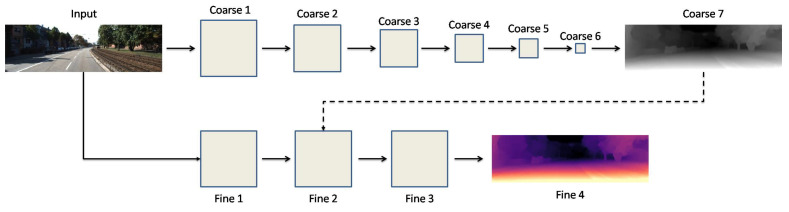
Developed network structure by Eigen et al. [9].

The SL approach for DE needs pixel-wise ground truth DI [114]. The SL procedure applies ground truth depth (GTD) to train a neural network as a regression model [83,115,116]. Eigen et al. [9] were pioneers in investigating DI to train a model using DL. They explained that their developed CNN-based network consists of two deep network stacks. Figure 10 presents a schematic illustration of the network structure proposed in [9]. As shown in Figure 10, the preparation of the input image occurred for both stacks. Additionally, the preparation of the output depth map of the first stack takes place to refine the depth map. The main responsibility of the second stack is to arrange obtained coarse depth predictions with the objects in the scene [9].

**Figure 11:**
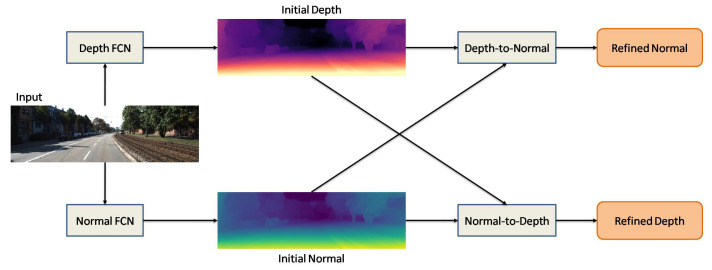
Developed geometric neural network by Qi et al. [37].

After Eigen’s investigation, different procedures were implemented to increase the precision of the estimated depth map (EDP). For example, Li et al. [117] developed a DL network applying conditional random fields (CRFs). They utilized a two-stage network for depth map estimation and refinement. In the first stage, a super-pixel technique on the input image is applied, and image patches are extracted around these super-pixels. In the second stage, CRFs are applied to refine the depth map by changing the super-pixel depth map to the pixel level. In order to extract an appropriate depth map, some approaches use geometric relationships. For example, Qi et al. [37] utilized two networks to estimate the depth map and surface normal from single images. Figure 11 depicts the developed network in [37]. These two networks enable the conversion of depth-to-normal and normal-to-depth and collaboratively increase the accuracy of the depth map and surface normal. Although their neural network can increase the accuracy of depth maps, for training, they require ground truth, including surface normal, which is hard to obtain. Ummenhofer et al. worked on developing a network to estimate depth maps using the structure from motion (SfM) technique. They corroborated that basic encoder–decoder architecture does not have sufficient capacity to process two input images simultaneously. Therefore, they developed a computer-based neural architecture that can extract optical flow, ego-motion, and a depth map from an image pair [38].

The dataset’s quality is an introductory section in SL systems, similar to methodology. Dos Santos et al. [118] paid enough attention to this challenge. They developed an approach to creating denser GTD maps from sparse LIDAR measurements via enhancing the valid depth pixels in depth images. They compared the obtained results of their trained model with both sparse GTD maps and denser GTD maps. They understood that the application of denser ground truth results yields increasing performance compared to sparse GTD maps. Ranftl et al. [119] developed an outstanding learning strategy that can involve various datasets to improve the efficiency of the MDE network. To prepare their dataset for three-dimensional movies, they applied stereo matching to conclude the depth of frames of these movies. Disparate unclear problems, including changing resolution and negative/positive disparity values, emerged during the creation of this dataset. According to the assistance of their developed procedures for incorporating multiple datasets, they achieved high precision with their model MDE problem. Recently, Sheng et al. [120] proposed a lightweight SL model with local–global optimization. They used an autoencoder network to predict the depth and used a local–global optimization scheme to realize the global range of scene depth.

### 6.2. Unsupervised Learning Approach

**Figure 12:**
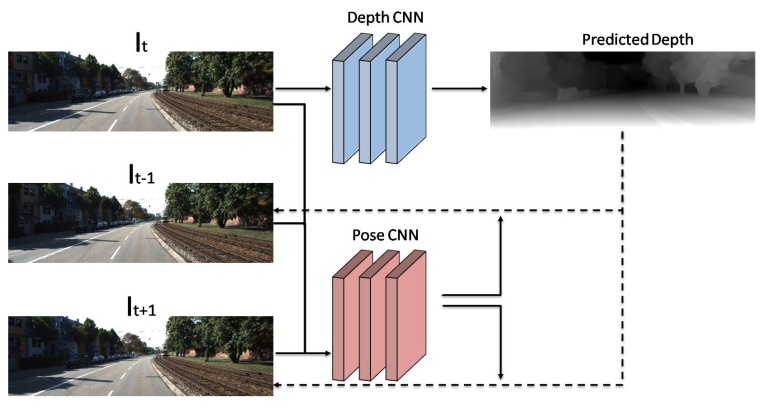
Developed network by Zhou et al. [16].

Increment of layers and trainable parameters in deep neural networks significantly increases the requirement for the train data, resulting in difficulty in achieving GTD maps. For this reason, UL approaches become an appropriate choice because unlabeled data is relatively easier to find [39,121,122]. Garg et al. [40] were the pioneers of developing a promising procedure to learn depth in an unsupervised fashion to remove the requirement of GTD maps. Up until now, developed UL approaches have applied stereo images, and thus, supervision and train loss depend intensely on image reconstruction. In order to train a depth prediction network, consecutive frames from a video may have great potential for application as supervision. Camera transformation estimation (pose estimation) between successive frames is the major challenge of this procedure, which results in extra complexity for the network. As illustrated in Figure 12, Zhou et al. [16] developed computer-based architecture to estimate depth map and camera pose simultaneously. As input, three successive frames are fed to the network. Pose CNN and Depth CNN estimate relative camera poses and a depth map from the first image.

**Figure 13:**
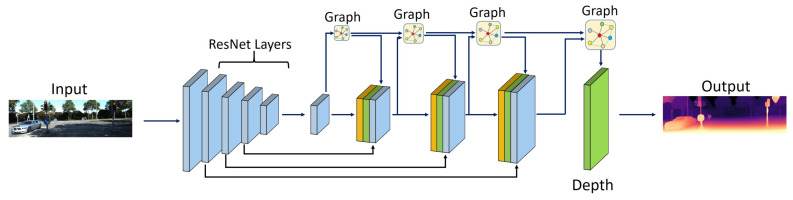
Developed network by Masoumian et al. [34].

In order to obtain greater accuracy in DE, some approaches have existed that possess the great potential of application to merge multiple self-supervision procedures into one. For instance, Godard et al. [83] applied MDE and estimated relative camera poses to build other stereoviews and contiguous frames in the video sequence. They added a pose network to their model to predict relative camera pose in adjacent frames. One of the crucial challenges towards using self-supervised approaches via video is occluded pixels. They applied minimum loss compared to the classical average loss to obtain non-occluded pixels, which is known as a significant improvement [7]. The improvement in the precision of UL approaches has motivated other investigators to modify knowledge distillation methods for the MDE problem. Pilzer et al. developed a system to adapt an unsupervised MDE network to the teacher–student learning framework by applying stereo image pairs to train a teacher network. Despite the promising performance of their student network, it was not as accurate as their teacher network [123]. Masoumian et al. [34] developed a multi-scale MDE based on a graph convolutional network. Their network consists of two parallel autoencoder networks: DepthNet and PoseNet. The DepthNet is an autoencoder composed of two parts: encoder and decoder; the CNN encoder extracts the feature from the input image, and a multi-scale GCN decoder estimates the depth map, as illustrated in Figure 13. PoseNet is used to estimate the ego-motion vector (i.e., 3D pose) between two consecutive frames. The estimated 3D pose and depth map are used to construct a target image.

### 6.3. Semi-Supervised Learning Approach

**Figure 14:**
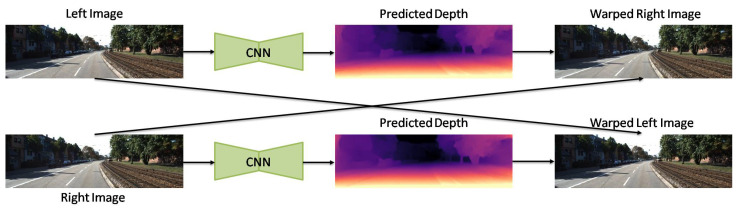
Components/inputs of the developed semi-supervised loss function by Kuznietsov et al. [26].

Compared to SL and UL approaches, few investigations have been conducted to study the performance of SSL methods for MDE. Apart from SL and UL approaches, Kuznietsov et al. [26] developed an SSL method by simultaneously applying supervised/unsupervised loss terms during training. Figure 14 demonstrates the components/inputs of the developed semi-supervised loss function in [26].

**Figure 15:**
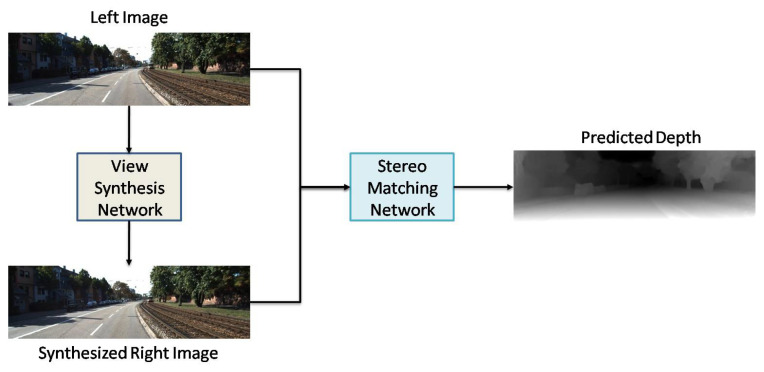
Components/inputs of developed semi-supervised loss function by Luo et al. [41].

In their approach, the estimated disparity maps (i.e., inverse depth maps) were used to rebuild left and right images via warping. Computation of unsupervised loss term took place by rebuilding the target images. Simultaneously, the calculation of the supervised loss term occurred by the estimated depth, and GTD maps [26]. Luo et al. [41] classified the MDE problem into two subdivisions and investigated them separately. Based on their procedure, the network requirement for labeled GTD data decreased. Additionally, they corroborated that the application of geometric limitations during inference may significantly increase the efficiency and the performance. Their proposed architecture is shown in Figure 15. Their developed architecture consists of two sub-networks, including view synthesis network (VSN) and stereo matching network (SMN). Their proposed VSN synthesizes the right image of the stereo pair via the left image. In SMN, simultaneous application of left and synthesized right images occurs in an encoder–decoder architecture pipeline to achieve a disparity map. In SMN, GTD maps are used to calculate the loss for estimated depth maps.

**Figure 16:**
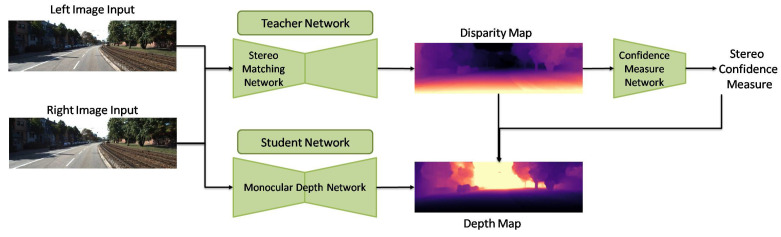
Developed network by Cho et al. [124].

Cho et al. [124] developed a novel teacher–student learning strategy to train an MDE network in an SSL approach. Their proposed procedure is demonstrated in Figure 16. They first introduced a stereo matching network with GT labeled data and permitted the teacher network to estimate depth from stereo pairs of an extensive unlabeled dataset. Then, they applied the aforementioned estimated depth maps/unlabeled dataset to train an optimized student network for MDE [124]. They also investigated the trade-off between the precision and the density of pseudo labeled depth maps. The density increases as the pixels in the depth map increase. They concluded the increment of the pseudo labeled depth maps’ precision by enhancing the density. Additionally, they reported that their MDE network achieved the greatest accuracy when the density of pseudo labeled depth maps was almost 80% [124].

## 7. Discussion

Due to the ability of humans to use theoretical-based information about the world, estimating depth maps from a single image may be easy for them [43]. Relying on the aforementioned fact, former investigations obtain MDE via mixing some old data, such as the communication between some geometric structures [17,28,125,126]. Due to the acceptable efficacy of image processing, CNN has illustrated a powerful capability to precisely predict dense depth maps from single images [9,127]. In recent years, numerous researchers have studied different types of cues of depth networks required for MDE according to four corroborated procedures, including MonoDepth, SfMLearner, Semidepth, and GCNDepth [7,16,26,34]. Deep neural networks are identified as a black box. In this black box, the supervised signals are applied to accelerate the learning process of some structural information for depth inference. The lack of sufficient datasets with ground truth due to their high economic cost can be considered one of the most critical DL problems. Table 6 aims to represent comprehensive information about the existing procedures based on their training data, supervised signals, and contributions.

### 7.1. Accuracy

To achieve high accuracy, several factors are involved. The first factor is using the supervised or unsupervised model. Our evaluation proves that supervised methods achieved higher accuracy than unsupervised and semi-supervised methods due to labeling the original ground truth. However, collecting a large dataset of monocular videos with accurate depth maps is a challenging task. Therefore, we can consider that unsupervised methods perform better than supervised methods if we neglect the slight difference in precision against the time for labeling data. Another factor is the frameworks of the developed networks. For instance, developing a DL model, such as graph convolution [34], 3D convolution [83], and 3D geometry constraint [84] outperforms other DL methods for DE. The last factor can be the loss of functions. There is some lack of information from monocular videos, such as scale inconsistency and scale ambiguity. One of the solutions for that is using semantic information and smooth loss to learn the scales. However, increasing the loss of functions will create more complicated networks and cause more computational time.

### 7.2. Computational Time

Computational times depend on the number of parameters of the whole network. The complex networks can predict high-quality and accurate depths, but this will cause them to not be considered in real-time applications due to the increased consumption power requirement. One of the best ways to reduce the computational time is to use pretrained models such as ResNet [142] or DenseNet [143] for feature extractions, and the model can focus only on the decoder part of the network. Table 7 represents the comparison of complex and lightweight models developed so far for monocular depth estimation based on the NYUDv2 dataset. As shown in Table 7, there is a kind of trade-off between the accuracy and the complexity of the models. The complex models [69] (e.g., [120] with 77 million parameters (params) and 186 G floating-point operations per second (FLOPs)) require higher computational time and with a large number of trained parameters; however, they give a more accurate depth estimation. On the contrary, lightweight models (e.g., [120] with 1.7 million params and 1.5 G FLOPs) require low computational time with a low number of trained parameters. Still, the accuracy is lower than complex models. In addition, the resolution of the resulted depth images is an essential key for increasing or decreasing the computational resources for the developed MDE models.

### 7.3. Resolution Quality

Computing a high-resolution DE is one of the main challenging tasks for researchers. Most of the current DE methods are suffering from this, and their results are not satisfied in reality. Based on the discussed training manners, it is evident that SL models [9,37,38] achieved higher quality resolution of depth maps than other models, such as UL [16,39,40]. SSL [26,41,42], because training the models with original ground truth helps the model to learn more accurately with higher quality resolution. However, one of the solutions for improving the resolution quality is to use super-resolution color images for training. However, this requires creating a new dataset which is expensive and time-consuming. In addition, the processing of high-resolution images/videos needs high computational resources that increase the cost, and obtaining high-resolution depth maps and computational resources is a trade-off.

### 7.4. Real-Time Inference

For using the MDE methods in industrial applications, it is very important that the model can perform in real time. There is a negative correlation between real-time performance and the complexity of the network, as shown in Table 7. Therefore, for better performance in real-time applications, lightweight MDE networks are required. However, researchers need to consider that lightweight networks sometimes reduce the accuracy and resolution of the predicted depth maps.

### 7.5. Transferability

Some networks are limited to working on the exact scenarios or environments, making them useless for other types of datasets. The transferability will make them more useful for different scenarios, cameras, and datasets. Training and testing the methods on different datasets, using domain adoption technology and 3D geometry, will improve the transferability of the models, and that will cause them to become more valuable in real life.

### 7.6. Input Data Shapes

As discussed earlier in Section 5, there are three types of input data: mono-sequence [16,33,34], stereo sequence [7,26], and sequence-to-sequence [35,36]. The mono-sequence input shapes models receive a single image as an input and provide a single output. These types are most commonly used in UL models. On the contrary, stereo-based models receive left and right pairwise images as inputs (i.e., one pair of images is used as a target image for unsupervised learning) and provide a single output as depth maps. These input shapes are mainly used for UL and SL models. The last type, sequence-to-sequence, is necessary for RNN models. These types receive a series of images as an input and provide a sequence of depth maps as an output. Due to the simplicity of the resources for mono-sequence and sequence-to-sequence models, which require a single camera compared to the stereo models, which require at least a pair of cameras, it is more economical to use mono-sequence or sequence-to-sequence models. On the other hand, sequence-to-sequence models require higher computational resources to train the model than mono-sequence models, since they need to process a sequence of images. Therefore, the most suitable models regarding low cost and computational resources are mono-sequence models.

### 7.7. Future Study

The current DL methods [34,83,148] have achieved the best performance so far. However, there is still no unit network that can predict a depth with high accuracy and resolution using low computational resources and without needing the actual ground truth. Therefore, the future study can create lightweight networks working on limited-memory devices without reducing the quality and resolution of predicted depth. In addition, the developed models should achieve higher accuracy under UL models to remove the original ground truth from training and create a self-adaption network for 3D reconstruction. Currently, the main challenges of MDE are that most MDE approaches depend on high-resolution images and large-size DL models with a high number of trained parameters that help predict depth maps with high accuracy. However, these models cannot be worked in real-time applications because they require high computational time and resources. On the contrary, lightweight networks are more useful for real-time applications and can be executed on devices with limited resources. However, reducing the networks’ complexity will significantly degrade the results’ quality and accuracy. Therefore, there is still a gap and limitation in this area to be discovered and solved.

Accurate real-depth annotations are difficult to acquire, needing special and expensive devices such as a LIDAR sensor. Self-supervised DE methods try to overcome this problem by processing video or stereo sequences, which may not always be available. Therefore, for DE, the researchers need to cope with the issue of domain adaption that will help train a monocular depth estimation model using a fully-annotated source dataset and a non-annotated target dataset. Additionally, although the MDE networks can be trained on an alternative dataset to overcome the dataset scale problem, the trained models cannot generalize to the target domain due to the domain discrepancy. For instance, there is no general MDE network that can still correctly predict the depth maps from day and night or indoor and outdoor images. In addition, most advanced MDE methods fail to predict accurate depth maps with adverse weather conditions (fogs, sunny, snow, etc.). Therefore, the future study requires a complete dataset to include day and night or indoor and outdoor images with different weather conditions.

## 8. Conclusions

DL techniques possess great potential to predict depth from monocular images. Implementation of depth prediction from monocular images is possible using an efficacious DL network structure and a dataset appropriate for the technique applied in learning. This paper presented a comprehensive overview of the contribution of this growing area of science in deep-learning-based MDE. Hence, the authors made an effort to review the state-of-the-art investigations on MDE from disparate aspects, including data input types, training manner and SL, UL, and SSL approaches combined with the application of different datasets and evaluation indicators. Finally, we highlight valuable opinions related to accuracy, computational time, resolution quality, real-time inference, transferability, and input data shapes, opening new horizons for future research. This paper demonstrates that the networks could train for various representation problems. In future perspectives, the architecture of DL models has to be improved to enhance the precision and reliability of the proposed networks and decline their inference time. Additionally, MDE networks have brilliant potential to be used in autonomous vehicles if high reliability is obtained. In addition, they must have the capability to output real-time depth maps.

## Figures and Tables

**Table 1:** Comprehensive to the related recent surveys in MDE in terms of six parameters; “TM”: training manner, “ACC”: accuracy, “CT”: computational time, “RQ”: resolution quality, “RTI”: real-time inference, “TRAN”: transferability, “IDS”: input data shapes.

Title	Year	TM	ACC	CT	RQ	RTI	TRAN	IDS
Deep-Learning-Based Monocular Depth Estimation Methods [17]	2020	✓	✓	✓				
Monocular Depth Estimation Based on Deep Learning [43]	2020	✓	✓			✓	✓	
Deep Learning for Monocular Depth Estimation [15]	2020	✓			✓			
Towards Real-Time Monocular Depth Estimation for Robotics [44]	2021	✓	✓	✓		✓		
Outdoor Monocular Depth Estimation [45]	2022							
Ours	2022	✓	✓	✓	✓	✓	✓	✓

**Table 2:** Comprehensive information about the quantitative results of the SL, SSL, and UL algorithms investigated on the KITTI dataset.

		Lower Better				Higher Better		
Method	Training Pattern	Abs-Rel	Sq-Rel	RMSE	RMSE-Log	δ<1.25	$\delta <1.25^{2}$	$\delta <1.25^{3}$
Bhat [69]	SL	0.058	0.190	2.360	0.088	0.964	0.995	0.999
Wang [70]	SL	0.088	0.245	1.949	0.127	0.915	0.9984	0.996
Patil [71]	SL	0.102	0.655	4.148	0.172	0.884	0.966	0.987
BTS [72]	SL	0.059	0.241	2.756	0.096	0.956	0.993	0.998
DepthNet [35]	SL	0.137	1.019	5.187	0.218	0.809	0.928	0.971
Kuznietsov [73]	SL	0.122	0.763	4.815	0.194	0.845	0.957	0.987
Monodepth [7]	SSL	0.148	1.344	5.927	0.247	0.803	0.922	0.964
SemiSup [26]	SSL	0.113	0.741	4.621	0.189	0.803	0.960	0.986
GMS [74]	SSL	0.143	2.161	6.526	0.222	0.850	0.939	0.972
GAN [75]	SSL	0.119	1.239	5.998	0.212	0.849	0.940	0.976
DepthGAN [76]	SSL	0.152	1.388	6.016	0.247	0.789	0.918	0.965
MonoRes [18]	SSL	0.111	0.867	4.714	0.199	0.864	0.954	0.979
Hints [77]	SSL	0.112	0857	4.807	0.203	0.862	0.952	0.978
SfMLearner [16]	UL	0.208	1.768	6.958	0.283	0.678	0.885	0.957
Vid2Depth [33]	UL	0.163	1.240	6.220	0.250	0.762	0.916	0.968
GeoNet [78]	UL	0.155	1.296	5.857	0.233	0.793	0.931	0.973
Struct2Depth [79]	UL	0.141	1.036	5.291	0.215	0.816	0.945	0.979
CC [80]	UL	0.140	1.070	5.326	0.217	0.826	0.941	0.975
LearnK [81]	UL	0.128	0.959	5.232	0.212	0.845	0.947	0.976
DualNet [82]	UL	0.121	0.837	4.945	0.197	0.853	0.955	0.982
Monodepth2 [83]	UL	0.115	0.882	4.701	0.190	0.879	0.961	0.982
FeatDepth [84]	UL	0.104	0.729	4.481	0.179	0.893	0.965	0.984
GCNDepth [34]	UL	0.104	0.720	4.494	0.181	0.888	0.965	0.984

**Table 3:** Comprehensive information about the quantitative results of the DL algorithms investigated on the NYU-V2 dataset.

		Lower Better				Higher Better		
Method	Training Pattern	Abs-Rel	Sq-Rel	RMSE	RMSE-Log	δ<1.25	$\delta <1.25^{2}$	$\delta <1.25^{3}$
DeepV2D [86]	SL	0.061	0.094	0.403	0.026	0.956	0.989	0.996
VNL [87]	SL	0.113	0.034	0.364	0.054	0.815	0.990	0.993
Fast-MVSNet [88]	SL	0.551	0.980	3.241	0.243	0.816	0.915	0.939
DORN [28]	SL	0.138	0.051	0.509	0.653	0.825	0.964	0.992
BTS [72]	SL	0.110	0.066	0.392	0.142	0.885	0.978	0.994
GASDA [89]	SSL	1.356	1.156	0.963	1.223	0.765	0.897	0.968
DnD [90]	SSL	0.213	0.320	2.360	0.084	0.761	0.889	0.932
DenseDepth [91]	SSL	0.093	0.589	4.170	0.171	0.886	0.965	0.986
SharpNet [19]	UL	0.139	0.047	0.495	0.157	0.888	0.979	0.995
MonoRes [18]	UL	1.356	1.156	0.694	1.125	0.825	0.965	0.967
DepthComple [92]	UL	0.842	0.760	5.880	0.233	0.863	0.921	0.972
Packnet-SfM [93]	UL	2.343	1.158	0.887	1.234	0.821	0.945	0.968
Monodepth2 [83]	UL	2.344	1.365	0.734	1.134	0.826	0.958	0.979

**Table 4:** Comprehensive information about the quantitative results of the DL algorithms investigated on the Make3D dataset.

Method	Training Pattern	Abs_Rel	Sq_Rel	RMSE	$\log_{10}$
Karsch [98]	SL	0.428	5.079	8.389	0.149
Liu [99]	SL	0.475	6.562	10.05	0.165
Laina [100]	SL	0.204	1.840	5.683	0.084
SfMLearner [16]	UL	0.383	5.321	10.47	0.478
DDVO [101]	UL	0.387	4.720	8.090	0.204
Monodepth2 [83]	UL	0.322	3.589	7.417	0.201
Jia [102]	UL	0.289	2.423	6.701	0.348
GCNDepth [34]	UL	0.424	3.075	6.757	0.107

**Table 5:** A summary of depth estimation public datasets.

Dataset	Sensors	Annotation	Type	Scenario	Images	Resolution	Year
KITTI [63]	LIDAR	Sparse	Real	Driving	44 K	1024×320	2013
NYU-V2 [106]	Kinect V1	Dense	Real	Indoor	1449	640×480	2012
Cityscapes [94]	Stereo Camera	Disparity	Real	Driving	5 K	1024×2048	2016
Make3D [96]	Laser Scanner	Dense	Real	Outdoor	534	2272×1704	2008
DIODE [103]	Laser Scanner	Dense	Real	In/Outdoor	25.5 K	768×1024	2019
Middlebury 2014 [104]	DSLR Camera	Dense	Real	Indoor	33	2872×1984	2014
Driving Stereo [105]	LIDAR	Sparse	Real	Driving	182 K	1762×800	2019

**Table 6:** Comprehensive information about the applied procedures in the deep learning of MDE.

Ref	Training Set	SL	SSL	UL	Major Contribution
Mousavian et al. [128]	RGB + Depth	✓			Multi-task (semantic + depth)
Jung et al. [129]	RGB + Depth	✓			Adversarial learning, global-to-local
Mayer et al. [64]	RGB + Depth	✓			Multi-task (optical flow + depth)
Laina et al. [100]	RGB + Depth	✓			Residual learning, BerHu loss
Kendall et al. [130]	Stereo sequences + Depth	✓			End-to-end learning
Fu et al. [28]	RGB + Depth	✓			Ordinal regression
Facil et al. [131]	RGB + Depth	✓			Multi-scale convolution
Wofk et al. [132]	RGB + Depth	✓			Lightweight network
Garg et al. [40]	Stereo sequences		✓		Image reconstruction, CNN
Chen et al. [133]	RGB + Relative depth annotations		✓		The wild scene dataset
Godard et al. [7]	Stereo sequences		✓		Left–right consistency
Kuznietsov et al. [26]	Stereo sequences + LIDAR		✓		Direct image alignment
Ramirez et al. [74]	Stereo sequences + Semantic label		✓		Semantic prediction
Pilzer et al. [76]	Stereo sequences		✓		Cycled generative network
Aleotti et al. [75]	Stereo sequences		✓		Generative adversarial network
He et al. [134]	Stereo sequences + LIDAR		✓		Sparse optimization
Fei et al. [135]	Stereo sequences + IMU + Semantic label		✓		Physical information
Li et al. [136]	Stereo sequences		✓		Absolute scale recovery
Zhao et al. [89]	Stereo sequences + Synthesized Depth		✓		Domain adaptation
Wu et al. [137]	Mono-sequences+LIDAR		✓		Attention mechanism
Zhou et al. [16]	Mono-sequences			✓	Ego-motion framework
Wang et al. [138]	Stereo sequences			✓	Multi-task (optical flow + depth)
Zhan et al. [39]	Stereo sequences			✓	Deep feature reconstruction
Chen et al. [139]	Mono-sequences			✓	Connecting flow, depth, and camera
Gordon et al. [81]	Mono-sequences			✓	Camera intrinsic prediction
Li et al. [140]	Mono-sequences			✓	Sequential adversarial learning
Almalioglu et al. [141]	Mono-sequences			✓	Generative adversarial network
Godard et al. [83]	Mono-sequences			✓	Left–right consistency
Shu et al. [84]	Mono-sequences			✓	Feature metric
Masoumian et al. [34]	Mono-sequences			✓	Graph multi-layer

**Table 7:** Comparison of complex and lightweight models based on the NYUDv2 dataset.

Group	Method	Resolution	FLOPs	Params	REL	RMS	RMSE-Log	δ<1.25	$\delta <1.25^{2}$	$\delta <1.25^{3}$
Complex	Hu et al. [144]	228 × 304	107G	67M	0.130	0.505	0.057	0.831	0.965	0.991
	Chen et al. [145]	228 × 304	150G	258M	0.111	0.420	0.048	0.878	0.976	0.993
	Yin et al. [87]	384 × 384	184G	90M	0.105	0.406	0.046	0.881	0.976	0.993
	Lee et al. [72]	416 × 544	132G	66M	0.113	0.407	0.049	0.871	0.977	0.995
	Bhat et al. [69]	426 × 560	186G	77M	0.103	0.364	0.044	0.902	0.983	0.997
Light Weight	Wofk et al. [132]	224 × 224	0.75G	3.9M	0.162	0.591	-	0.778	0.942	0.987
	Nekrasov et al. [146]	480 × 640	6.49G	2.99M	0.149	0.565	-	0.790	0.955	0.990
	Yin et al. [87]	338 × 338	15.6G	2.7M	0.135	-	0.060	0.813	0.958	0.991
	Hu et al. [147]	228 × 304	14G	1.7M	0.138	0.499	0.059	0.818	0.960	0.990
	Sheng et al. [120]	228 × 304	1.5G	8.2M	0.135	0.488	0.057	0.831	0.966	0.991

## Data Availability

Not applicable.

## Abbreviations

The following abbreviations are used in this manuscript:

DE	Depth Estimation
MDE	Monocular Depth Estimation
BDE	Binocular Depth Estimation
MVDE	Multi-View Depth Estimation
DL	Deep Learning
DI	Depth Information
CNN	Convolutional Neural Network
SL	Supervised Learning
UL	Unsupervised Learning
SSL	Semi-Supervised Learning
GTD	Ground Truth Depth
EDM	Estimated Depth Map
VO	Visual Odometry
